# Absence of Association between Behavior Problems in Childhood and Hypertension in Midlife

**DOI:** 10.1371/journal.pone.0167831

**Published:** 2016-12-09

**Authors:** Sadiq M. Saad, Gurch Randhawa, Dong Pang

**Affiliations:** Institute for Health Research, University of Bedfordshire, Luton, United Kingdom; TNO, NETHERLANDS

## Abstract

**Background:**

It is known that behavior in childhood is associated with certain physical and mental health problems in midlife. However, there is limited evidence on the role of childhood behavior problems in the development of hypertension in adulthood. The present study aimed to examine whether behavior problems in childhood influenced the risk of hypertension in midlife in the United Kingdom 1958 birth cohort.

**Methods:**

The 1958 British birth cohort comprised 17,638 individuals born in the first week of March 1958 in the United Kingdom. Behavior problems were assessed at 7, 11, and 16 years of age by parents and teachers. At age 45, blood pressure was measured and hypertension was recorded if blood pressure was ≥140/90 mm Hg or if the participants were informed by their health professionals that they had high blood pressure. Behavioral information was reported according to the Rutter Children’s Behaviour Questionnaire (RCBQ) and the Bristol Social Adjustment Guide (BSAG). Odds ratios (ORs) and 95% confidence intervals (CIs) were calculated to examine behavior problems in childhood in relation to hypertension at 45 years of age according to logistic regression analysis, with adjustment for sex, social class in childhood and adulthood, childhood cognition, birth weight, gestational age at birth, body mass index (BMI), smoking, alcohol consumption, and physical activity.

**Results:**

Behavior problems reported by parents at 7, 11, and 16 years were not associated with hypertension in midlife (OR, 0.93; 95% CI, 0.81, 1.07; OR, 0.95; 95% CI, 0.81, 1.11; OR, 0.98; 95% CI, 0.85, 1.12, respectively). Similarly, teacher-reported behavior problems at 7, 11, and 16 years were not associated with hypertension in midlife (OR, 0.92; 95% CI, 0.72, 1.18; OR, 0.92; 95% CI, 0.84, 1.02; OR, 1.03; 95% CI, 0.92, 1.15, respectively). Further separate analyses showed similar results for males and females.

**Conclusion:**

There is no association between behavior problems in childhood and hypertension in midlife.

## Introduction

Hypertension is a major risk factor for cardiovascular disease [1,2]. Previous studies suggested that early life is a critical period in the development of hypertension and related diseases in later life [3–8]. Studies have demonstrated that childhood psychological factors, such as cognition and mental ability, are associated with high blood pressure in adulthood [7,8]. The type A behavior pattern in adulthood has been shown to be associated with coronary heart disease [9,10]. However, there is limited evidence for the role of behavior problems in childhood in the development of hypertension. A previous regional study on a Scottish birth cohort found no significant association between behavior problems in childhood and hypertension in midlife [11]. In this study, childhood behavior problems were measured at a single age point (11 years) by a single assessor (teacher). This method may have been inadequate to capture the trajectory of behavior changes in childhood and behavior problems at home. In addition, there is increasing evidence for the influence of behavioral disturbances in childhood on mental health [12,13], chronic widespread pain [14], and injury and death [15,16] in later life. The purpose of the present study is to establish whether behavior problems in childhood are associated with hypertension in midlife, based on data from a national cohort (the 1958 British birth cohort). In particular, we investigated childhood behavior, which was reported by both parents and teachers at multiple time points in childhood (at ages 7, 11, and 16 years).

## Materials and Methods

### Participants

The 1958 British birth cohort comprises 17,638 infants born during the first week of March 1958 in England, Scotland, and Wales, accounting for 98% of live births in that period. In addition, 920 individuals born elsewhere who immigrated to the United Kingdom before the age of 16 years and who were born within the same period (March 3 to 9, 1958) were included in the cohort (*n* = 18,558). The cohort was followed up from birth in 1958 to the age of 45 years in 2003, with data obtained at 7, 11, and 16 years in childhood and at 23, 33, 42, and 45 years in adulthood. During the first three follow-up periods in childhood, information was obtained from parents, the local authorities, and classroom teachers. In the survey at 45 years, 1,245 cohort members had died and 1,300 had emigrated. Of the remaining 16,013 participants, 3,004 were not contacted and 3,622 were nonrespondents. A total of 9,377 participants took part in the biomedical survey at 45 years [17]. The 45-year sample was shown to be broadly representative of the original cohort with respect to sex ratio, childhood social class, housing tenure, and physical and maternal factors. However, those with behavior problems at 7 years were under-represented in the 45-year sample (10.8% vs. 13% in the 7-year sample) [17]. The present study has 80% power to detect a twofold increased risk of hypertension among those with behavior problems compared with those without behavior problems at a 5% significance level (two-sided).

### Blood pressure at 45 years

Blood pressure measurements were taken from participants at 45 years during a biomedical survey by trained research nurses. Blood pressure was measured in a quiet room, and the measurements were done with an Omron 705CP automated sphygmomanometer (Omron, Tokyo, Japan), which was validated according to international protocol [18]. Blood pressure measurements were repeated three times for each participant while the participant was seated and resting for 5 minutes after each measurement. Both systolic and diastolic blood pressures were recorded. The participants were asked if they had been informed by a doctor that they had high blood pressure and/or if they were taking any medication for high blood pressure. In this study, hypertension was defined as a systolic blood pressure of 140 mm Hg or higher or a diastolic blood pressure of 90 mm Hg or higher according to World Health Organization guidelines [19,20]. Any current use of medication for high blood pressure by an individual was also classified as hypertension. Hypertension was treated as a binary (yes/no) variable in the statistical analysis.

### Behavior problems in childhood

Reports of childhood behavior were obtained from parents (mainly mothers) and schoolteachers. The parents were requested to give an account of childhood behavior at 7, 11, and 16 years using scale A of the Rutter Children’s Behaviour Questionnaire (RCBQ), while the teachers assessed childhood behavior at school using Rutter scale B at 16 years and the Bristol Social Adjustment Guide (BSAG) at 7 and 11 years. The Rutter scale A used at 7 years consists of 14 items (0–28 scale), and the Rutter scale A used at 11 years consists of 18 items (0–36 scale). The Rutter scale B used at 16 years consists of 26 items (0–52 scale). Each item on both scales (A and B) was recoded according to a uniform coding protocol as follows: 0 = does not apply/never, 1 = applies somewhat/sometimes, or 2 = certainly applies/frequently, in relation to the child. The scores of individual items on each scale were added up to obtain a total behavior score for each follow-up [14]. Inter-rater reliability and retest reliability were reported as 0.64 and 0.74 for the Rutter scale A and 0.72 and 0.89 for the Rutter scale B [21,22], respectively. The BSAG, which was completed by the teachers, lists 150 descriptions of behavior or attitudes at school that might apply to the child. These descriptions were grouped into syndromes as follows: unforthcomingness (timidity and fear), withdrawal, depression, anxiety for acceptance by adults, hostility toward adults, “writing off” of adults and adult standards, anxiety for acceptance by children, restlessness, “inconsequential” behavior, miscellaneous symptoms, and miscellaneous nervous symptoms [23]. The scores for the different categories were added up to obtain a total behavior score (0–99 scale) [14]. The participants were divided into three childhood behavior groups according to their scores at 7, 11, and 16 years. Those who scored below the 80th percentile were considered to have normal behavior, those who scored above the 95th percentile were considered to have severe behavior problems, and those with scores between the 80th and 95th percentiles were considered to have mild-to-moderate behavior problems [14]. The original behavior scores were not comparable across the three age points because they were measured by different rating scales. In addition, we assessed persistent behavior problems at 7, 11, and 16 years. We defined persistent behavior problems as behavior problems at 16 years and at least one other childhood age. The RCBQ has been shown to be a valid and reliable tool for assessing behavior problems in both clinical and nonclinical settings [24]. In addition, the classification of behavior problems used in the present study was recommended in the user manual of the 1958 British birth cohort and was previously used by Pang and colleagues in assessing the relationship between behavior problems and chronic widespread pain [14].

### Covariates

Some potential confounding factors were considered, including social class, birth weight, and adult health behaviors. Childhood social class was defined according to the Registrar General’s classification of father’s occupation in 1958 as class I (professional), class II (managerial and technical), class IIInm (skilled nonmanual), class IIIm (skilled manual), class IV (semiskilled), and class V (unskilled) [25]. When information for 1958 was not available, the father’s occupation when the cohort member was 7 years of age was used. Social class at age 42 was based on the cohort member’s current or most recent occupation at that age according to the same classification that was used at birth. A childhood general ability test was used as a measure of childhood cognition. At 11 years, the participants took an age-appropriate general ability test at school, which had verbal (0–40 scale) and nonverbal (0–40 scale) components. Birth weight of the study participants was recorded in pounds and ounces and converted to grams. Low birth weight was defined as less than 2,000 g [26], and gestational age at birth less than 37 weeks was considered to be preterm. The participants’ height was measured with a stadiometer. We calculated body mass index (BMI) as the weight in kilograms divided by the square of the height in meters [27]. Health behavior variables included in this study were smoking, excessive alcohol consumption, and physical activity. This information was collected from the participants by a computer-assisted self-administered interview. The participants were categorized at age 42 as current smokers (one or more cigarettes per day), former smokers, or never smokers. Data on alcohol consumption were also collected. Study participants were asked to fill in questions that were developed for the detection of alcoholism with the CAGE (Cut-down, Anger, Guilt, and Eye-opener) alcohol questionnaire. The Alcohol Use Disorders Identification Test [28,29] was used to record frequency of drinking (daily, weekly, monthly, or never) and number of drinks per day (0, 1–2, 3–4, 5–6, or ≥7). In addition, the participants were asked about their exercise frequency at age 42 years from a list of sport and leisure activities (<2–3 times per month, once a week, 2–3 times per week, or 4–7 times per week).

### Statistical analysis

The association between behavior problems and hypertension was assessed by logistic regression. The association was expressed as odds ratios (ORs) and 95% confidence intervals (CIs). In the regression analysis, hypertension was treated as a binary dependant variable. Separate analyses were performed for teacher- and parent-reported behavior at 7, 11, and 16 years. Sex, social class, birth weight, gestational age at birth, BMI, and health behaviors (smoking, alcohol consumption, and physical activity) were taken into account in the regression analysis (models 1, 2, and 3). We ran a separate model for each behavior report. A trend test was performed to examine the exposure–response relationship between behavior problems and hypertension by treating the behavior variable as a continuous variable. Data on blood pressure at 45 years were available for 9,270 participants. Initially, the analysis was carried out on participants with complete information on behavior at 7, 11, 16 years and blood pressure as well as other variables, such as sex, birth weight, gestational age at birth, social class, BMI, smoking, alcohol consumption, and physical activity. To test the robustness of our findings, ORs were re-estimated by multiple imputation to allow for missing data. Specifically, childhood behavior problems, hypertension, and potential confounders were imputed using multiple imputation by chain equations as described by van Buuren and colleagues [30]. Five copies of the original dataset imputed for missing data were created, and ORs were calculated according to Rubin’s rule [31]. The results of the multiple imputation method were similar to those of the complete case method. In this paper, we present the results of the multiple imputation method. All analyses were performed with Stata 13 [32].

### Ethical approval

Ethical approval for the original birth study was obtained from the South-East Multicentre Research Ethics Committee. All participants’ records and information were anonymized and de-identified prior to analysis.

## Results

The behavior of 14,923, 14,156, and 11,670 participants was assessed at 7, 11, and 16 years, respectively. At age 45, the blood pressure of 9,376 participants was measured. Table 1 shows the prevalence of hypertension at midlife according to parents’ and teachers’ reporting of behavior problems in childhood. Around 23% of the participants at 45 years had hypertension. The prevalence of hypertension among men was higher than that among women (32.4% vs. 13.2%).

**Table 1:** Prevalence of hypertension in midlife by behavior problems in childhood.

		All subjects		Men		Women	
Behavior		N	%	N	%	N	%
All (*n* = 9,376)		2,105	22.81	1,494	32.42	611	13.22
**Parent-reported behavior**^**a**^							
	**Age 7**						
	Normal	1,343	22.82	971	33.39	372	12.50
	Mild-to-moderate	336	22.64	229	30.21	107	14.74
	Severe	56	22.49	33	25.38	23	19.33
	**Age 11**						
	Normal	1,250	22.59	878	33.02	372	12.94
	Mild-to-moderate	327	23.49	245	32.41	82	12.89
	Severe	59	25.76	42	33.07	17	16.67
	**Age 16**						
	Normal	1,288	23.24	923	32.92	365	13.34
	Mild-to-moderate	224	21.81	141	30.39	83	14.74
	Severe	69	27.06	50	40.32	19	14.50
**Teacher-reportedbehavior**^**b**^							
	**Age 7**						
	Normal	1,550	22.31	1,069	32.51	481	13.14
	Mild-to-moderate	297	24.32	229	32.16	68	13.36
	Severe	80	27.59	66	33.00	14	15.56
	**Age 11**						
	Normal	1,562	22.18	1,072	32.22	490	13.19
	Mild-to-moderate	338	26.10	268	34.31	70	13.62
	Severe	68	25.37	58	32.58	10	11.11
	**Age 16**						
	Normal	1,268	22.11	889	31.98	379	12.83
	Mild-to-moderate	237	25.62	159	32.19	78	18.10
	Severe	49	24.14	41	33.88	8	9.76

Hypertension is defined as blood pressure ≥140/90 mm Hg.

^a^Rutter scale A: <80% of the score, no behavior problems; top 5% of the score, severe behavior problems; between the 80th and 95th percentiles, mild-to-moderate behavior problems.

^b^Rutter scale B at 16 years, Bristol Social Adjustment Guide (BSAG) scale at 7 and 11 years: <80% of the score, no behavior problems; top 5% of the score, severe behavior problems; between the 80th and 95th percentiles, mild-to-moderate behavior problems.

Table 2 shows the association between childhood behavior problems and hypertension at 45 years. The results of the fully adjusted analysis did not show a significantly increased risk of hypertension with increased severity of childhood behavior problems (model 3) as reported by parents (OR, 0.93; 95% CI, 0.81, 1.07; OR, 0.95; 95% CI, 0.81, 1.11; OR, 0.98; 95% CI, 0.85, 1.12, at 7, 11, and 16 years, respectively) or teachers (OR, 0.92; 95% CI, 0.72, 1.18; OR, 0.92; 95% CI, 0.84, 1.02; OR, 1.03; 95% CI, 0.92, 1.15, at 7, 11, and 16 years, respectively). When we compared individuals with scores indicating behavior problems (in particular, severe behavior problems) with those with normal behavior scores reported by parents, the results did not indicate a significantly increased risk of hypertension at 45 years among those with childhood behavior problems (OR, 0.95; 95% CI, 0.81, 1.11; OR, 0.90; 95% CI, 0.56, 1.45; OR, 1.08; 95% CI, 0.73, 1.61, at 7, 11, and 16 years, respectively). Similar results were shown for teacher-reported behavior problems at 7, 11, and 16 years. We further examined the association between persistent behavior problems and hypertension. The results did not show a significant association between persistent behavior problems in childhood and hypertension at age 45.

**Table 2:** Behavior problems in childhood in relation to hypertension in midlife.

		Model 1	Model 2	Model 3
Behavior		OR (95% CI)	OR (95% CI)	OR (95% CI)
**Parent-reported behavior**^**a**^				
	**Age 7**			
	Normal	1	1	1
	Mild-to-moderate	0.96 (0.80, 1.16)	0.94 (0.78, 1.13)	0.92 (0.77, 1.00)
	Severe	0.96 (0.75, 1.23)	0.90 (0.70, 1.15)	0.87 (0.67, 1.13)
	Trend	0.97 (0.85, 1.11)	0.94 (0.83, 1.08)	0.93 (0.81, 1.07)
	**Age 11**			
	Normal	1	1	1
	Mild-to-moderate	0.99 (0.88, 1.12)	0.96 (0.86, 1.08)	0.94 (0.82, 1.08)
	Severe	0.97 (0.63, 1.51)	0.92 (0.59, 1.43)	0.90 (0.56, 1.45)
	Trend	1.01 (0.90, 1.12)	0.96 (0.84, 1.10)	0.95 (0.81, 1.11)
	**Age 16**			
	Normal	1	1	1
	Mild-to-moderate	0.92 (0.74, 1.15)	0.88 (0.71, 1.08)	0.88 (0.71, 1.09)
	Severe	1.18 (0.83, 1.69)	1.08 (0.77, 1.53)	1.08 (0.73, 1.61)
	Trend	1.06 (0.94, 1.19)	0.98 (0.86, 1.11)	0.98 (0.85, 1.12)
	**Persistent behavior problems at ages 7, 11, and 16**			
	Normal behavior	1	1	1
	Persistent behavior problems	1.02 (0.87, 1.21)	0.96 (0.81, 1.14)	0.94 (0.78, 1.19)
**Teacher-reported behavior**^**b**^				
	**Age 7**			
	Normal	1	1	1
	Mild-to-moderate	1.00 (0.81, 1.26)	0.91 (0.70, 1.19)	0.90 (0.68, 1.18)
	Severe	1.03 (0.71, 1.48)	0.91 (0.58, 1.42)	0.88 (0.55, 1.39)
	Trend	1.01 (0.91, 1.12)	0.93 (0.74, 1.19)	0.92 (0.72, 1.18)
	**Age 11**			
	Normal	1	1	1
	Mild-to-moderate	1.08 (0.98, 1.20)	0.99 (0.87, 1.14)	0.96 (0.84, 1.09)
	Severe	0.98 (0.76, 1.26)	0.87 (0.68, 1.11)	0.81 (0.62, 1.05)
	Trend	1.03 (0.95, 1.11)	0.96 (0.88, 1.05)	0.92 (0.84, 1.02)
	**Age 16**			
	Normal	1	1	1
	Mild-to-moderate	1.15* (1.03, 1.29)	1.07 (0.94, 1.22)	1.02 (0.88, 1.19)
	Severe	1.22 (0.97, 1.54)	1.11 (0.85, 1.44)	1.06 (0.79, 1.43)
	Trend	1.13 (1.05, 1.20)	1.06 (0.97, 1.16)	1.03 (0.92, 1.15)
	**Persistent behavior problems at ages 7, 11, and 16**			
	Normal behavior	1	1	1
	Persistent behavior problems	1.15 (0.92, 1.43)	1.03 (0.78, 1.35)	0.99 (0.77, 1.26)

Model 1: basic model, adjusted for sex.

Model 2: adjusted for sex, birth weight, gestational age at birth, childhood cognition and social class at birth.

Model 3: adjusted for sex, birth weight, gestational age at birth, childhood cognition, social class at birth, smoking at 42 years, BMI, social class at 42 years, physical activity, and alcohol consumption.

^*a*^Rutter scale A: <80% of the score, no behavior problems; top 5% of the score, severe behavior problems; between the 80th and 95th percentiles, mild-to-moderate behavior problems.

^*b*^Rutter scale B at 16 years, Bristol Social Adjustment Guide (BSAG) scale at 7 and 11 years: <80% of the score, no behavior problems; top 5% of the score, severe behavior problems; between the 80th and 95th percentiles, mild-to-moderate behavior problems.

*p < 0.05.

Table 3 shows the relationship between behavior problems at ages 7, 11, and 16 and hypertension among men. In the fully adjusted analysis, there was a negative association between parent-reported behavior problems at 7 years and hypertension, with a 20% decrease in the risk of hypertension among men with mild-to-moderate behavior problems in childhood (OR, 0.80; 95% CI, 0.67, 0.96) compared with those without behavior problems (model 3). There were no significant associations between parent-reported severe behavior problems at 11 and 16 years and hypertension in men (OR, 0.85; 95% CI, 0.43, 1.69; OR, 1.06; 95% CI, 0.80 1.41, respectively). Similar results were obtained for teacher-reported severe behavior problems at 11 and 16 years and hypertension in men.

**Table 3:** Behavior problems in childhood in relation to hypertension in midlife among men.

		Model 1	Model 2	Model 3
Behavior		OR (95% CI)	OR (95% CI)	OR (95% CI)
**Parent-reported behavior**^***a***^				
	**Age 7**			
	Normal	1	1	1
	Mild-to-moderate	0.82* (0.68, 0.98)	0.80* (0.67, 0.96)	0.80* (0.67, 0.96)
	Severe	0.78 (0.55, 1.12)	0.72 (0.50, 1.04)	0.71 (0.50, 1.00)
	**Age 11**			
	Normal	1		1
	Mild-to-moderate	1.01 (0.87, 1.17)	0.98 (0.85, 1.23)	0.97 (0.83, 1.13)
	Severe	0.90 (0.50, 1.62)	0.85 (0.48, 1.53)	0.85 (0.43, 1.69)
	**Age 16**			
	Normal	1	1	1
	Mild-to-moderate	0.86 (0.60, 1.24)	0.81 (0.57, 1.16)	0.82 (0.55, 1.20)
	Severe	1.17 (0.94, 1.44)	1.06 (0.86, 1.32)	1.06 (0.80, 1.41)
	**Persistent behavior problems at ages 7, 11, and 16**			
	Normal behavior	1	1	1
	Persistent behavior problems	0.97 (0.76, 1.25)	0.91 (0.70, 1.19)	0.93 (0.69, 1.25)
**Teacher-reported behavior**^***b***^				
	**Age 7**			
	Normal	1	1	1
	Mild-to-moderate	0.96 (0.79, 1.17)	0.87 (0.69, 1.10)	0.86 (0.68, 1.00)
	Severe	1.00 (0.67, 1.49)	0.89 (0.56, 1.41)	0.88 (0.55, 1.39)
	**Age 11**			
	Normal	1	1	1
	Mild-to-moderate	1.07 (0.92, 1.23)	0.98 (0.82, 1.17)	0.95 (0.80, 1.13)
	Severe	1.03 (0.80, 1.33)	0.91 (0.69, 1.21)	0.84 (0.64, 1.11)
	**Age 16**			
	Normal	1	1	1
	Mild-to-moderate	1.09 (0.94, 1.28)	1.01 (0.85, 1.21)	0.97 (0.76, 1.25)
	Severe	1.24 (0.88, 1.76)	1.12 (0.75, 1.67)	0.86 (0.50, 1.50)
	**Persistent behavior problems at ages 7, 11, and 16**			
	Normal behavior	1	1	1
	Persistent behavior problems	1.11 (0.85, 1.45)	0.98 (0.69, 1.39)	0.94 (0.68, 1.32)

Model 1: basic model.

Model 2: adjusted for birth weight, gestational age at birth, childhood cognition and social class at birth.

Model 3: adjusted for birth weight, gestational age at birth, childhood cognition, social class at birth, smoking at 42 years, BMI, social class at 42 years, physical activity, and alcohol consumption.

^*a*^Rutter scale A: <80% of the score, no behavior problems; top 5% of the score, severe behavior problems; between the 80th and 95th percentiles, mild-to-moderate behavior problems.

^*b*^Rutter scale B at 16 years, Bristol Social Adjustment Guide (BSAG) scale at 7 and 11 years: <80% of the score, no behavior problems; top 5% of the score, severe behavior problems; between the 80th and 95th percentiles, mild-to-moderate behavior problems.

*p < 0.05.

Table 4 shows the relationship between behavior problems at ages 7, 11, and 16 and hypertension among women. After adjustment for known confounders in the fully adjusted analysis (model 3), neither parent- nor teacher-reported childhood behavior problems were significantly associated with hypertension in women.

**Table 4:** Behavior problems in childhood in relation to hypertension in midlife among women.

		Model 1	Model 2	Model 3
Behavior		OR (95% CI)	OR (95% CI)	OR (95% CI)
**Parent-reported behavior**^**a**^				
	**Age 7**			
	Normal	1	1	1
	Mild-moderate	1.36* (1.02, 1.80)	1.32* (1.00, 1.73)	1.23 (0.94, 1.60)
	Severe	1.47 (0.77, 2.83)	1.40 (0.75, 2.62)	1.32 (0.73, 2.40)
	**Age 11**			
	Normal	1		1
	Mild-to-moderate	0.95 (0.72, 1.26)	0.92 (0.71, 1.19)	0.89 (0.68, 1.16)
	Severe	1.17 (0.59, 2.32)	1.10 (0.55, 2.16)	1.02 (0.54, 1.94)
	**Age 16**			
	Normal	1	1	1
	Mild-to-moderate	1.04 (0.74, 1.47)	1.10 (0.70, 1.49)	1.02 (0.68, 1.54)
	Severe	1.20 (0.50, 2.90)	1.13 (0.47, 2.70)	1.13 (0.47, 2.68)
	**Persistent behavior problems at ages 7, 11, and 16**			
	Normal behavior	1	1	1
	Persistent behavior problems	1.14 (0.86, 1.51)	1.09 (0.82, 1.43)	0.99 (0.75, 1.30)
**Teacher-reported behavior**^**b**^				
	**Age 7**			
	Normal	1	1	1
	Mild-to-moderate	1.13 (0.62, 2.05)	1.01 (0.54, 1.87)	0.99 (0.51, 1.91)
	Severe	1.12 (0.68, 2.20)	0.95 (0.47, 1.95)	0.84 (0.40, 1.75)
	**Age 11**			
	Normal	1	1	1
	Mild-to-moderate	1.13 (0.88, 1.45)	1.02 (0.76, 1.39)	0.98 (0.72, 1.34)
	Severe	0.74 (0.42, 1.57)	0.65 (0.35, 1.19)	0.63 (0.35, 1.16)
	**Age 16**			
	Normal	1	1	1
	Mild-to-moderate	1.30 (0.99, 1.69)	1.22 (0.97, 1.53)	1.16 (0.91, 1.48)
	Severe	1.13 (0.58, 2.19)	1.04 (0.53, 2.05)	1.04 (0.53, 2.06)
	**Persistent behavior problems at ages 7, 11, and 16**			
	Normal behavior	1	1	1
	Persistent behavior problems	1.25 (0.96, 1.62)	1.16 (0.86, 1.56)	1.11 (0.80, 1.55)

Model 1: basic model.

Model 2: adjusted for birth weight, gestational age at birth, childhood cognition and social class at birth.

Model 3: adjusted for birth weight, gestational age at birth, childhood cognition, social class at birth, smoking at 42 years, BMI, social class at 42 years, physical activity, and alcohol consumption.

^*a*^Rutter scale A: <80% of the score, no behavior problems; top 5% of the score, severe behavior problems; between the 80th and 95th percentiles, mild-to-moderate behavior problems.

^*b*^Rutter scale B at 16 years, Bristol Social Adjustment Guide (BSAG) scale at 7 and 11 years: <80% of the score, no behavior problems; top 5% of the score, severe behavior problems; between the 80th and 95th percentiles, mild-to-moderate behavior problems.

*p < 0.05.

## Discussion

This study found no association between behavior problems in childhood and hypertension in midlife. Behavior problems reported by parents and teachers at any of three ages in childhood, as well as behavior problems that persisted for at least two ages were not associated with midlife hypertension.

Our findings are consistent with those of Von Stumm and colleagues, who reported no association between childhood behavior problems and adult high blood pressure in a Scottish birth cohort study [11]. Although this was a prospective, longitudinal cohort study, all health information, including blood pressure, was self-reported. Furthermore, behavior was assessed by one person (teacher) and at one age point in childhood (11 years). In contrast, blood pressure measurements in the present study were taken three times by nurses, and behavior was assessed by two persons at multiple ages in childhood (7, 11, and 16 years).

Before we draw any conclusions, however, we need to rule out the possibility of artifactual null association that may occur as a result of attrition over time. Although Atherton and colleagues (2008) have shown that the 45-year sample is largely representative of the original cohort [17], attrition of the sample over 45 years may have introduced bias. For instance, individuals with childhood behavior problems at age 7 are under-represented in the 45-year sample [17]. In this study, potential attrition bias due to missing data was accounted for in all analyses by using the multiple imputation method. The study has 80% power to detect a twofold increased risk of hypertension among those with behavior problems in childhood compared with those without behavior problems in childhood at a 5% significance level.

The reasons for associations between childhood behavior and adult mortality or morbidity [12–16] are largely unknown. It is possible that there are common causes of childhood behavior and adult disease, such as socioeconomic position and cognition. An alternative explanation is that childhood behavior predicts later health-related behavior, which in turn affects the risk of adult disease. However, the present study does not support any association between childhood behavior and hypertension in midlife.

The main limitation of our study is the relatively young age of the participants at the time of assessment of outcome (45 years). The risk of hypertension increases with increasing age [33], and hypertension is more prevalent among individuals over 50 years of age [34]. One possible explanation for the lack of association between childhood behavior problems and adult hypertension in the present study may be that the midlife cohort in our study had yet to reach the average age of diagnosis of hypertension. The results of the present study may reflect a true lack of association between childhood behavior problems and hypertension in midlife. Further research with longer follow-up would be helpful to clarify whether this conclusion holds for individuals in later life.

Nevertheless, our study has some advantages. First, our analysis was based on a large, population-based sample, which was prospectively followed up for more than four decades. Second, information on behavior was prospectively collected, thus eliminating recall bias, which is often encountered in retrospective studies. Furthermore, the assessment of behavior for each study subject was carried out by two raters (parent and teacher) at three age points in childhood (7, 11, and 16 years). This was necessary because of the nature of changeable child behavior. Analyses of parent-reported and teacher-reported behavior problems yielded consistent results. Third, unlike classical cohort studies, in which the participants may have been born over a wide range of dates at the beginning of the study, the present study is free of confounding effects due to age because all participants were born in the same week.

## Conclusion

There is no association between childhood behavior problems and hypertension at midlife. Although childhood behavior problems have been increasingly reported to be associated with a range of health problems, such as mental illness, widespread pain, injury, and others, there does not appear to be a major concern about an increased risk of hypertension among individuals with a history of childhood behavior problems.
